# Perception of evidence-based practice and the professional environment of Primary Health Care nurses in the Spanish context: a cross-sectional study

**DOI:** 10.1186/1472-6963-12-227

**Published:** 2012-07-31

**Authors:** Susana González-Torrente, Jordi Pericas-Beltrán, Miguel Bennasar-Veny, Rosa Adrover-Barceló, José M Morales-Asencio, Joan De Pedro-Gómez

**Affiliations:** 1 Primary Health Care, Balearic Islands Health Service, Palma, Spain; 2 Nursing Department, Evidence Based Clinical Practice and Knowledge Transfer Research Group Members, Balearic Islands University, Carretera de Valldemossa Km 7.5, 07122, Palma, Islas Baleares, Spain; 3 Faculty of Health Sciences, University of Málaga, Málaga, Spain

## Abstract

**Background:**

The study of the factors that encourage evidence-based clinical practice, such as structure, environment and professional skills, has contributed to an improvement in quality of care. Nevertheless, most of this research has been carried out in a hospital context, neglecting the area of primary health care. The main aim of this work was to assess the factors that influence an evidence-based clinical practice among nursing professionals in Primary Health Care.

**Methods:**

A multicentre cross-sectional study was designed, taking the 619 Primary Care staff nurses at the Balearic Islands’ Primary Health Care Service, as the study population. The methodology applied consisted on a self-administered survey using the instruments *Evidence-Based Practice Questionnaire (EBPQ)* and *Nursing Work Index (NWI)*.

**Results:**

Three hundred and seventy seven surveys were received (60.9% response rate). Self-assessment of skills and knowledge, obtained 66.6% of the maximum score. The *Knowledge/Skills* factor obtained the best scores among the staff with shorter professional experience. There was a significant difference in the *Attitude* factor (p = 0.008) in favour of nurses with management functions, as opposed to clinical nurses.

Multivariate analysis showed a significant positive relationship between NWI and level of evidence-based practice (p < 0,0001).

**Conclusions:**

Institutions ought to undertake serious reflection on the lack of skills of senior nurses about Evidence-Based Clinical Practice, even when they have more professional experience. Leadership emerge as a key role in the transferral of knowledge into clinical practice.

## Background

Evidence-Based Clinical Practice (EBCP) is known to improve the quality of health care, making it cost-efficient while improving clinical results [1]. But the barriers for transferring research into clinical practice [2-4] challenge this process, which, in their final state, would culminate in what Davis called ‘the adoption process’ [5].

Dijkstra (2006) synthesized some barriers and facilitators, as well as interventions, to improve the implementation of new knowledge, identifying that they depend, basically, on factors associated to the professionals, the organisation they work for and their management policies [6]. Grimshaw (1999) and Grol (2001) have already proposed the complexity of the variables involved in the transferral of knowledge into clinical practice [7,8].

It is worrying to observe how, along the route that lies between the production of knowledge and the clinical decision of professionals, there is a progressive decrease in knowledge, in favour of beliefs, opinions, etc. [9]*.* Several studies have attempted to analyse this phenomenon from different perspectives, such as the influence of knowledge management [9,10], attitudes, values or training in the process of knowledge transferral into clinical practice [11,12]. Barriers perceived by professionals concerning the use of research into clinical practice have been studied [13-15], as well as the lack of support of health organisations towards EBCP [16].

Most of the studies conducted on the implementation of EBCP have focused on the area of Hospital Care (HC) [17-24] and frequently on highly specialized units, such as intensive care [25]. The emergence of Magnet Hospitals in USA [26,27] promoted the study of environmental factors that influence nursing practice and the subsequent development of instruments as the Nursing Work Index (PES-NWI) [17,28,29].

On the other hand, some questionnaires have been created for analysing factors that promote or prevent evidence based practice, like the Evidence Based Practice Questionnaire (EBPQ) [30]. But there is a paucity of studies analysing these factors in the PHC context, even though the increasing concern about disseminating knowledge in this environment [14,28,31].

In order to diagnose the different elements that influence the impact of organisational climate in nursing practice, [Gershon et al. (2004) 28] identified 12 instruments , where NWI is one of the most used [32]. This instrument was initially designed by [Kramer and Hafner in 1989 31] and since its initial version, has been refined successively. The most disseminated versions are the one made by [Aiken et al (2000) 17], and the PES-NWI, validated by Lake (2002), which has shown the greatest explanatory parsimony [33]. In Spain, the NWI-R has been used occasionally, but without a previous validation process [19,33], unlike the PES-NWI, which has undergone a validation process in our context [34,35].

The field of professional skills for EBCP has been less studied and, as a result, existing instruments have been more scarce [36-38] and only a few of them have been validated carefully [38]. In 2006, Upton and Upton reported the EBPQ [30], which has been adapted and validated into the Spanish context [39,40].

The aim of this study was to assess the factors that influence EBCP in the Balearic Islands’ Health Service. For this, the objectives of the study were to describe the EBCP patterns in primary health care nurses, the analysis of possible differences related to features of the staff, such gender, age, experience and main practice (management or clinical) and to evaluate the potential influence of the practice environment where they work.

## Methods

### Design and setting

A multicentre cross-sectional study was designed, taking the whole Primary Care staff nurses at the Balearic Islands’ Public Health Care Service as the study population. The study was conducted in 2009, among the 619 nurses distributed between 57 health centres (HC).

Nurses received a personalized letter presenting the project and containing the two instruments, along with a request to participate, and a commitment for guarantying anonymity and the compliance with Organic Law 15/1999 on Protection of Details of a Personal Nature. Once answered, the questionnaires were introduced into a closed envelope and handed to the different members of the research team.

Ethical approval for the study was granted by the Balearic Clinical Research Ethical Committee (CEIC-IB). All nurses participating in the study were informed that they could abandon it at any time.

### Data collection

In order to assess the practice environment, we used the NWI questionnaire validated in Spain [41]. The instrument contains 31 items, grouped in 5 factors: 1) *Nurse participation in issues related to HC*; 2) *Foundation of quality of nursing care*; 3) *Capacity, leadership and support to nurses by nurse managers*; 4) *Dimension of nursing staff and adequacy of human resources* and 5) *Relationship between physicians and nurses*.

To assess knowledge, use and attitudes of professionals towards EBCP, we used the EBPQ questionnaire, also validated in Spain [39]. This instrument is made up of 24 items structured in three factors: 1) *Practice*; 2) *Attitude and knowledge*; and 3) *Skills of professionals in EBCP*.

Other variables measured were the professional category (clinical nurses and nurse managers) and number of years in professional service.

### Data analysis

The strategy of analysis comprised an examination of the descriptive data of the sample, bivariate analysis with parametric and non-parametric tests, depending on the nature of the distributions (correlation, ANOVA, *Kruskall-Wallis*, chi squared). In order to go further into the objective of the study, a multivariate regression model was developed to analyse the effect of the environments on evidence-based practice of professionals. Age, gender, years of professional practice (grouped by 0–2 years; >2–10 years; >10–20 years; >20 years) and type of practice (clinical or management) and the overall NWI score were taken as independent variables. As a dependent variable, the overall EBPQ score was used. For this analysis, independence was checked using *Durbin-Watson* statistics, homocedasticity through the association between residuals and typified prognosis, normality, using a histogram of typified residuals and linearity, with partial regression graphs.

All analyses were conducted using the statistical package PASW 18.0 and confidence levels were set at 95% (p = 0.05).

## Results

### Respondents

Of the 619 nurses in primary care practice, 377 responded to the questionnaire (60.9%). The respondents consisted of 324 female (86.2%) and 52 male (13.8%). The population had a mean professional experience of 20.9 years. The overall mean age was 44.5 years DE: 10.8 (CI 95% 43.4–45.6). The mean age for men (52) was 43.8 years, DE: 10.6 (CI 95%: 40.8–46.7) and for women (310) 44.6 years, DE: 10.9 (CI 95%: 43.4–45.8). Most of them (98.1%) held a 3 years Universitary Degree in Nursing, while the remaining 1.9% had studied an additional Degree in other disciplines.

Three hundred and twenty two (89.2%) were clinical nurses and 39 (7.9%) were nurse managers. Concerning to years of experience as nurses, 12 (3.3%) had been working for less than 2 years; 77 (21.0%) between 2 and 10 years; 84 (22.9%) between 10 and 20 years and the remaining 194 (52.9%), over 20 years.

From this population, 213 (56.6%) had children and 75.4% of them (72.0% men and 76.0% women) lived with a stable partner.

The overall assessment and a breakdown of the different factors (both for the EBPQ and the PES-NWI) can be seen in Table 1.

**Table 1:** Assessment of total and different factors in the EBPQ and NWI

	**Score**	**DE**	**CI 95%**
EBPQ total	112.7	20.8	(110.6–114.8)
Practice	27.7	7.5	(26.0–28.5)
Attitude	21.1	3.9	(20.7–21.5)
Knowledge/Skills	63.9	13.3	(62.6–65.3)
NWI total	80.4	15.1	(78.9–82.0)
Participation	21.7	5.7	(21.1–22.3)
Foundation	26.2	5.5	(25.7–26.8)
Management support	15.6	3.8	(15.2–16.0)
Adequate staff	8.8	3.1	(8.5–9.1)
Physician/nurse	8.1	2.4	(7.8–8.4)

377 participants responded to both questionnaires.

### Assessment of questionnaires

Regarding the level of professional competence perceived by nurses in order to develop EBCP (measured with the EBPQ questionnaire) - significant intergroup differences were found in the overall score, depending on the number of years of professional experience (p = 0.018). With regard to the three factors that make up the EBPQ (*Practice*, *Attitude* and *Knowledge/Skills*), significant differences in the *Knowledge/Skills* factor were maintained (p = 0.023). It is worth noting that the professionals with shorter experience obtained the best scores (Table 2).

**Table 2:** Assessment of the total EBPQ and of its factors according to the different groups of number of years in professional practice

**Factor (Vmax)**^**a**^	**Mean**	**CI at 95 %**		**DE**	** *p* **
		**Lower limit**	**Upper limit**		
Practice (42)					
Intergroups	27.6	26.8	28.4	7.5	0.065
0 to 2 years	32.7	29.8	35.6	4.6	
2 to 10 years	28.3	26.9	29.7	6.1	
10 to 20 years	26.9	24.9	28.8	8.9	
>20 years	27.3	26.3	28.4	7.5	
Attitude (28)					
Intergroups	21.0	20.6	21.5	4.0	0.094
0 to 2 years	22.3	20.1	24.5	3.5	
2 to 10 years	21.2	20.4	22.0	3.7	
10 to 20 years	21.8	21.0	22.5	3.5	
>20 years	20.6	20.0	21.2	4.2	
Knowledge/Skills (98)					
Intergroups	63.8	62.5	65.2	13,4	0,023
0 to 2 years	72.3	66.4	78.2	9,3	
2 to 10 years	66.1	63.1	69.2	13,5	
10 to 20 years	63.9	61.4	66.5	11,9	
>20 years	62.4	60.4	64.33	13,9	
EBPQ total (168)					
Intergroup	112.5	110.3	114.6	20.8	0.018
0 to 2 years	127.3	117.4	137.0	15.4	
2 to 10 years	115.6	111.1	120.1	19.8	
10 to 20 years	112.5	108.2	116.9	20.1	
>20 years	110.3	107.3	113.3	21.3	

Anova factor test was applied.

^a^Vmax: maximum value of the factor of the questionnaire.

There was a better score for nurses with management functions (supervision and coordination), compared to clinical nurses, in the *Attitude* factor (p = 0.008) .

The comparison of the PES-NWI among the different strata derived from years of professional experience, showed a significant intergroup difference (p < 0.027) in the *Physician/Nurse relationship* factor. Despite the lack of statistical significance, a propensity towards obtaining a better score in the groups with lesser experience could be seen, especially in the *0 to 2 years* group (Table 3).

**Table 3:** Assessment of the total NWI and of its factors according to the different groups of number of years in professional practice

**Factor (Vmax)**^**a**^	**Mean**	**CI at 95 %**		**DE**	** *p* **
		**Lower limit**	**Upper limit**		
Participation (36)					
Intergroup	21.6	21.0	22.2	5.7	0.657
0 to 2 years	23.3	20.3	26.4	4.8	
2 to 10 years	22.0	20.7	23.4	6.1	
10 to 20 years	21.5	20.4	22.7	5.4	
>20 years	21.4	21.1	22.3	5.8	
Foundation (40)					
Intergroup	26.1	25.6	26.7	5.5	0.157
0 to 2 years	29.3	26.0	32.5	5.1	
2 to 10 years	26.2	24.9	27.5	5.7	
10 to 20 years	25.5	24.3	26.6	5.4	
>20 years	26.2	25.4	27.0	5.4	
Manager support (20)					
Intergroup	15.5	15.1	15.9	3.8	0.260
0 to 2 years	16.1	13.3	18.9	4.4	
2 to 10 years	16.2	15.4	17.0	3.5	
10 to 20 years	15.6	14.8	16.4	3.6	
>20 years	15.2	14.6	15.8	4.0	
Adequacy of staff (16)					
Intergroup	8.8	8.4	9.1	3.2	0.777
0 to 2 years	8.8	7.2	10.3	2.5	
2 to 10 years	8.4	7.8	9.1	2.9	
10 to 20 years	8.8	8.1	9.4	3.1	
>20 years	8.9	8.4	9.4	3.3	
Physician/nurse relationship (12)					
Intergroup	8.1	7.9	8.4	2.4	0.027
0 to 2 years	8.3	7.9	10.6	2.2	
2 to 10 years	8.2	7.6	8.7	2.4	
10 to 20 years	8.6	8.1	9.1	2.3	
>20 years	7.8	7.5	8.1	2.4	
NWI total (124)					
Intergroup	80.2	78.6	81.7	15.0	0.415
0 to 2 years	86.7	78.3	95.0	13.2	
2 to 10 years	80.1	77.8	84.2	14.1	
10 to 20 years	79.9	76.7	83.2	15.0	
>20 years	79.5	77.3	81.7	15.4	

Anova factor test was applied.

^a^Vmax: maximum value of the factor of the questionnaire.

In the analysis of the overall score according to the type of professional category, nurses who carried out management functions had significantly higher scores (p = 0.004), in three factors: *Participation in issues related to the centre*, *Foundation of quality nursing care* and *Dimension of nursing staff and adequacy of human resources*. No differences were found in the other factors: *Capacity*, *Leadership* and *Nursing support by nurse managers* and *Relationships between physicians and nurses* (Table 4).

**Table 4:** Assessment of the total NWI and of its factors according to professional category

**Factor (Vmax)**^**a**^	**Mean**	**CI at 95 %**		**DE**	** *p* **
		**Lower limit**	**Upper limit**		
Participation (36)					
Clinical nurse	21.3	20.6	21.9	5.7	0.001
Nurse manager	24.5	23.0	26.1	4.9	
Foundation (40)					
Clinical nurse	25.9	25.2	26.5	5.6	0.010
Nurse manager	28.3	26.9	29.7	4.4	
Manager support (20)					
Clinical nurse	15.4	15.0	15.9	3.9	0.116
Nurse manager	16.5	15.7	17.3	2.5	
Adequacy of staff (16)					
Clinical nurse	8.7	8.3	9.0	3.2	0.037
Nurse manager	9.8	8.7	10.8	3.3	
Physician/nurse relationship (12)					
Clinical nurse	8.2	7.9	8.4	2.4	0.309
Nurse manager	7.7	7.0	8.5	2.2	
NWI total (124)					
Clinical nurse	79.4	77.7	81.0	15.2	0.004
Nurse manager	86.8	82.9	90.7	11.9	

322 clinical nurses and 39 nurse managers responded validly.

^a^Vmax: maximum value of the factor of the questionnaire.

EBPQ and PES-NWI showed no difference among female or male nurses.

Multivariate analysis detected a significant relationship among NWI, years in practice (age was eliminated due to its collinearity with the number of years in practice) and the level of evidence-based practice, with an explanatory capacity of 30% (Table 5).

**Table 5:** Multivariate analysis

	**Non-standard coefficients**	**Typified coefficients**	**Sig**	**CI at 95 %**	
				**Lower limit**	**Upper limit**
(Constant)	77.050		0.000	57.284	96.816
NWI total	0.337	0.236	0.000	0.188	0.487
Gender	−2.302	−0.039	0.460	−8.428	3.824
Category	1.171	0.036	0.491	−2.170	4.512
Years of practice	−0.733	−0.377	0.004	−1.230	−0.236

Multiple regression was carried out with the EBPQ as a dependent variable and the ones in the first column as predictor variables.

## Discussion

The response rate obtained in our study is higher than that obtained in other studies which percentages of 40% [42] or even lower [43]. However, they are very similar, as far as the proportion of men and women (1 man for every 6 women) and the mean age of the nurses surveyed (over 40 years) is concerned. Regarding the professional experience (mean = 20.9 years) our study obtains similar figures to those achieved by [Gerrish et al. (2011) 43]in England , but, on the whole, they are higher than those in other similar studies carried out in the United States, where the mean number of years worked is 10 [42,44]. With respect to the academic level it is difficult to compare with other countries like the United Kingdom, United States or Canada, where most of the studies have been conducted and nurses have a long tradition in professional development, including the possibility of access to a doctorate.

In relation to the results of the EBPQ questionnaire, we observed some remarkable results, as the higher scores obtained among nurses with shorter professional experience. Thus, the group that obtains the highest score is the one made up of nurses in their first two years of clinical practice, and the one that obtains the lowest score is the group 10–20 years. These data point out the existence of a paradoxical and deep perception of lack of competence with respect to the use of EBCP, among experienced nurses. This result mismatches with other studies conducted outside Spain using EBPQ, where the greater the number of years of experience, the higher the score obtained by nurses in the *Daily practice* factor [42], or no significant differences were observed [45].

These contradictory results could be explained by three factors: the lack of a professional career which encourages nurses to make progress on their own development, the absence of specialisation in Community Nursing in Spain until recently, and the common fact in all the Spanish territory of highly experienced hospital nurses moving to Primary Health Care to conclude the end stage of their professional career in this context, where they are not expert.

Perhaps, the youngest nurses tend to consider themselves more capable for developing EBCP because of their recent university experience, where these issues are studied, and the continuation of relations with Faculty staff that could support them in the processes of seeking and interpretation of evidence [46].

With respect to the significant difference in the *Attitude* factor of the EBPQ in favour of nurses with management functions as opposed to clinical ones, this could be due to the greater commitment and motivation of nurses who accept to perform as nursing coordinators in health centres. Despite this position has few rewards, it is generally accepted by nurses with a high degree of professional development [47].

The NWI factor which obtains the highest score is *Management support* (78% of the possible maximum score), reflecting that nurses consider the support from nursing coordinators a key factor in achieving the objectives related to EBCP. Several studies have identified managers, not only as a key factor for the generation and implementation of EBCP, but also for the creation of a good research environment [48-50]. In other studies, nurses have declared the need for a mentor to guide them along the search and implementation of evidence [45,46,49]. Shirey (2006), in reference to the creation of magnet Hospitals, states the key role of leadership and how it can be fostered with appropriate training [49]. In this respect, there are several publications concerning experiences of EBCP implementation based on teaching and training nurse coordinators [48,50,51].

Also, in the PES-NWI factor *Foundation of quality nursing care,* there is a significant difference in favour of nurse managers. Gerrish et al. (2011), in a study carried out among 855 hospital and primary health care nurses in England to implement EBCP in their environments, consider that holding a master degree would be recommended for those nurses who lead the implementation process [43]. Therefore, it is patent the essential role of leadership in the process of implementation evidence into clinical practice [52].

In the light of the NWI results in the *Participation* factor, the nurses in our study seem to feel that they are not too involved in issues concerning the centre, although once again, the managers score higher in this factor. As afore mentioned, it is of vital importance for the generation and implementation of EBN to create a work environment that encourage the participation and involvement of all nurses in providing evidence-based quality care [53,54]. Kramer and Schmalenberg (2008), stated that in magnet hospitals, (where a good working environment encourages nursing staff recruitment and quality of care), what managers understand as a good working environment is not necessarily the same for the nursing staff [55]. We believe that this clarification is also applicable to the implementation of EBCP into PHC.

It seems realistic, given the structure of our PHC organisations, that nursing coordinators and managers feel more involved in the organisation than other nurses. This may be influenced by the fact that the design of information flow is pyramidal, and nursing coordinators have prior access to information, or they are frequently consulted about possible changes, and they are the ones who transmit this to the staff in their HC. In this same sense, there is a relevant study conducted in the Community of Madrid in which a generalised complaint concerning poor feedback by management to different PHC teams, reports how aims and priorities are not clear, teams are rarely informed about the expectations that are set and managers do not support, recognize or show enough gratitude for the good work carried out by the teams [56].

In the NWI, the total *Adequacy of the staff* factor obtains 55% of the maximum score, although compared with the rest of the factors it is the lowest valued one. The ratio of nurses per population in Spain is below the European standard [57]. It is well known that a shortage of nurses creates a loop of job dissatisfaction due to work overload, which results in a worse quality of care provided and it is an important barrier for implementing EBCP [44,54]. In studies conducted in the Spanish PHC environment, the need for training and adequacy of staff dimension is highlighted, emphasizing the imbalance between nursing staff and GPs, with poorer figures in nurses. Moreover, references are made to the health system working badly, by creating new positions and responsibilities for PHC without providing the parallel means to put them into practice, thus creating bad feeling between professionals and increasing stress [56,57]. Besides, Sancho-Viudes et al. (2002) in the PHC environment in Mallorca identified - as problems to be solved - the poor adequacy of staff dimension compared to the population to be cared for, the need for updating clinical guides and protocols, the lack of specific training in community nursing, and the subsequent need for a specialization in community nursing [47]. Kramer and Schmalenberg (2008), when they describe the requirements that must be met in order to generate a good climate in the work place, point out the perception of staff adequacy [55]. This difference in perception between nurse managers and staff nurses should alert us to the fact that managers may be slightly isolated from their nurses and their daily reality.

Squires et al. (2011) in a recent review concludes that, in all the studies examined, the attitude of nurses towards research is the only individual characteristic that is positively related to the use of research at work, although carrying out clinical sessions in the workplace, having higher qualifications, a professional career, a clinical specialization or a high job satisfaction are also important [58]. Even though the resolution of potential barriers that hinder EBCP is a necessary condition, this could be not enough to succeed, as other less objective or measurable factors such as values, motivation, environment or empathy are just as important or even more than the former [59]. A recent review carried out by Solomons and Spross (2011) concludes that many of the measurement scales used in the studies do not measure these aspects [60]. In another recent study, Levin piloted an EBCP implementation model among home visiting nurses in New York which took into account their beliefs regarding EBCP, group cohesion, productivity, job satisfaction and burnout due to staff changes, relating a greater implementation of EBCP with lower burnout and greater satisfaction with the work group [61].

## Conclusion

The findings from this study highlight the importance of organisational culture and context in achieving EBCP. This problem affects the relationships between institutions and nurses throughout many countries, as it is reflected in the literature, and it has been also corroborated in this study carried out in Spain.

The commitment of nurses and health managers at the highest level is required to promote a change in achieving EBCP, to assure the highest level of competence and effectiveness which should lead to improved patient outcomes.

It would be worth thinking about the need to create a nursing research friendly work environment, supportive towards knowledge transfer, by putting into practice what some authors call a culture change, which would involve an increase in communication and collaboration in both organisational and clinical decision making, a more active attitude towards research in the work place, and a change in attitudes and beliefs.

Furthermore, it would be highly desirable to improve the leadership skills for nurse coordinators, as they appear to be key agents in the implementation of a culture more favourable towards EBCP. On the other hand, these strategies should involve the whole PHC team, always taking into account the characteristics of the nurses, in such a way that they feel they are contributing and are useful, above all with highly experienced nurses, because of their key role in implementing EBCP.

## Limitations

This study has some limitations. It is a cross-sectional design that does not allow us to explore the directionality of the associations and, on the other hand, elements such as leadership - which appear to bear increasingly more weight in the adoption of evidence by professionals - have been explored tangentially. Moreover, in the sampling process, there was an under-representation of the group of professionals with 0 to 2 years’ experience, with 14% under the expected with respect to the size of this stratum in the different centres. This selection bias might have been caused by the selection criteria in order to respond to the survey, which excluded professionals with less than 6 months in their work place, and these nurses had worked mainly on short-term contracts and there are more job perspectives for novel nurses in hospitals than in PHC.

Balearic Islands is a geographically isolated territory and it is plausible that an own culture could determine some of these findings. Therefore, the generalisation of these results should be made carefully to other communities.

## Competing interests

All authors declare no competing interests.

## Author’s contributions

SGT was involved in the study conception and design, drafting and conducting the surveys among nurses. JPB was involved in the study conception and design, drafting and conducting the surveys among nurses. MBV and JMMA were involved in the study design as well as in drafting and critical revision of the manuscript. RAB was involved in the study conception and design and conducting the surveys among nurses. JDPG was involved in the study conception and design, drafting and critical revision of the critical revision of the manuscript. RAB was involved in the study conception and design and conducting the surveys among nurses. JDPG was involved in the study conception and design, drafting and critical revision of the manuscript, supervision and statistical analysis. All authors read and approved the final manuscript.

## Funding

This article is part of a research project financed by the Health Research Fund (PI 09/90512. Health Ministry) following a rigorous peer-reviewed funding process.

## Pre-publication history

The pre-publication history for this paper can be accessed here:

http://www.biomedcentral.com/1472-6963/12/227/prepub
